# Spectro-Temporal Processing in a Two-Stream Computational Model of Auditory Cortex

**DOI:** 10.3389/fncom.2019.00095

**Published:** 2020-01-22

**Authors:** Isma Zulfiqar, Michelle Moerel, Elia Formisano

**Affiliations:** ^1^Maastricht Centre for Systems Biology, Maastricht University, Maastricht, Netherlands; ^2^Department of Cognitive Neuroscience, Faculty of Psychology and Neuroscience, Maastricht University, Maastricht, Netherlands; ^3^Maastricht Brain Imaging Center, Maastricht, Netherlands

**Keywords:** auditory cortex, sound processing, dynamic neuronal modeling, temporal coding, rate coding

## Abstract

Neural processing of sounds in the dorsal and ventral streams of the (human) auditory cortex is optimized for analyzing fine-grained temporal and spectral information, respectively. Here we use a Wilson and Cowan firing-rate modeling framework to simulate spectro-temporal processing of sounds in these auditory streams and to investigate the link between neural population activity and behavioral results of psychoacoustic experiments. The proposed model consisted of two *core* (A1 and R, representing primary areas) and two *belt* (*Slow* and *Fast*, representing rostral and caudal processing respectively) areas, differing in terms of their spectral and temporal response properties. First, we simulated the responses to amplitude modulated (AM) noise and tones. In agreement with electrophysiological results, we observed an area-dependent transition from a temporal (synchronization) to a rate code when moving from low to high modulation rates. Simulated neural responses in a task of amplitude modulation detection suggested that thresholds derived from population responses in *core* areas closely resembled those of psychoacoustic experiments in human listeners. For tones, simulated modulation threshold functions were found to be dependent on the carrier frequency. Second, we simulated the responses to complex tones with missing fundamental stimuli and found that synchronization of responses in the *Fast* area accurately encoded pitch, with the strength of synchronization depending on number and order of harmonic components. Finally, using speech stimuli, we showed that the spectral and temporal structure of the speech was reflected in parallel by the modeled areas. The analyses highlighted that the *Slow* stream coded with high spectral precision the aspects of the speech signal characterized by slow temporal changes (e.g., prosody), while the *Fast* stream encoded primarily the faster changes (e.g., phonemes, consonants, temporal pitch). Interestingly, the pitch of a speaker was encoded both spatially (i.e., tonotopically) in *Slow* area and temporally in *Fast* area. Overall, performed simulations showed that the model is valuable for generating hypotheses on how the different cortical areas/streams may contribute toward behaviorally relevant aspects of auditory processing. The model can be used in combination with physiological models of neurovascular coupling to generate predictions for human functional MRI experiments.

## Introduction

The processing of sounds in primate auditory cortex (AC) is organized in two anatomically distinct streams: a *ventral* stream originating in areas located rostrally to the primary auditory core and projecting to the ventral regions of the frontal cortex, and a *dorsal* stream originating in areas located caudally to the primary core and projecting to dorsal frontal regions. Processing in these separate streams is hypothesized to underlie auditory cognition and has been linked respectively to specialized mechanisms of sound analysis for deriving semantic information (“what” processing) or processing sound location and sound movement (“where” processing) (Kaas et al., 1999; Romanski et al., 1999; Belin and Zatorre, 2000; Kaas and Hackett, 2000; Rauschecker and Tian, 2000; Tian et al., 2001; Arnott et al., 2004). Interestingly, the basic response properties (e.g., frequency tuning, latencies, temporal locking to the stimulus) of neurons in areas of dorsal and ventral auditory streams show marked differences (Rauschecker et al., 1996; Bendor and Wang, 2008; Oshurkova et al., 2008; Nourski et al., 2013, 2014), and differences have been reported even for neurons from areas within the same (dorsal) stream (Kuśmierek and Rauschecker, 2014). A consistent observation is that neurons in the rostral field, in comparison to primary and surrounding auditory areas, exhibit longer response latencies and narrower frequency tuning (Recanzone et al., 2000; Tian et al., 2001; Bendor and Wang, 2008; Camalier et al., 2012), whereas neurons in the caudal fields respond with shorter latencies, comparable to or even shorter than those in A1, and have broader frequency tuning (Recanzone et al., 2000; Kuśmierek and Rauschecker, 2014). How this organization of neuronal properties within AC contributes to the processing of spectro-temporally complex sounds remains unclear and poses an interesting question for computational endeavors (Jasmin et al., 2019).

Recent results of neuroimaging studies in humans have put forward the hypothesis that fine-grained spectral properties of sounds are analyzed optimally in ventral auditory regions, whereas fine-grained temporal properties are analyzed optimally in dorsal regions (Schönwiesner and Zatorre, 2009; Santoro et al., 2014). It is, however, unlikely that the neural processing of spectral and temporal properties of sounds is carried out through completely independent mechanisms. Several psychophysical phenomena such as pitch perception based on temporal cues (Houtsma and Smurzynski, 1990; Bendor et al., 2012) or the frequency dependence of amplitude modulation (AM) detection thresholds (Sek and Moore, 1995; Kohlrausch et al., 2000) suggest an interdependence between neural processing mechanisms for spectral and temporal properties.

Therefore, in this study, we aim to introduce a simple, stimulus-driven computational framework for modeling the spectral and temporal processing of sounds in AC and examine the role of the different processing streams. We use the firing rate model of Wilson and Cowan (Wilson Cowan Cortical Model, WCCM; Wilson and Cowan, 1972, 1973; Cowan et al., 2016) which simulates complex cortical computations through the modeling of dynamic interactions between excitatory and inhibitory neuronal populations. Over the years, WCCM has been successfully implemented for simulating neuronal computations in the visual cortex (Ermentrout and Cowan, 1979; Wilson and Kim, 1994; Wilson, 1997). More recently, WCCM has been applied to the AC as well to describe the propagation of activity in the interconnected network of cortical columns and to generate predictions about the role of spontaneous activity in the primary AC (Loebel et al., 2007), and the role of homeostatic plasticity in generating traveling waves of activity in the AC (Chrostowski et al., 2011). Furthermore, WCCM has been proposed for modeling stimulus-specific adaptation in the AC (May et al., 2015; Yarden and Nelken, 2017) and to generate experimentally verifiable predictions on pitch processing (Tabas et al., 2019), etc. While WCCMs are less detailed than models of interconnected neurons, they may provide a right level of abstraction to investigate functionally relevant neural computations, probe their link with psychophysical observations, and generate predictions that are testable using invasive electrocorticography (ECoG) as well as non-invasive electro- and magneto-encephalography (EEG, MEG) and functional MRI (fMRI) in humans.

Here, we used the WCCM to simulate the dynamic cortical responses (population firing rates) in the AC to both synthetic and natural (speech) sounds. After filtering from the periphery, the proposed model processes the spatiotemporally structured (i.e., tonotopic) input in two primary auditory *core* areas. The output of the core areas is then fed forward to two secondary auditory *belt* areas, which differ in terms of their processing of spectral and temporal information and thereby represent the dorsal and ventral auditory processing streams. In a number of simulations, we used this model to examine the coding of amplitude modulated (AM) broadband noise and tones using metrics derived from the electrophysiology (firing rate and temporal synchronization with the stimulus). We also simulated three psychoacoustic experiments to study the role of the multiple information streams that may underlie behavioral AM detection thresholds observed for noise (Bacon and Viemeister, 1985) and tones (Kohlrausch et al., 2000), as well as pitch perception with missing fundamental stimuli (Houtsma and Smurzynski, 1990). Lastly, we investigated the processing of speech stimuli in the model in order to generate predictions on how this cortical spectro-temporal specialization (represented by the four areas) may encode the hierarchical structure of speech.

## Materials and Methods

### Model Design and Architecture

**FIGURE 1:**
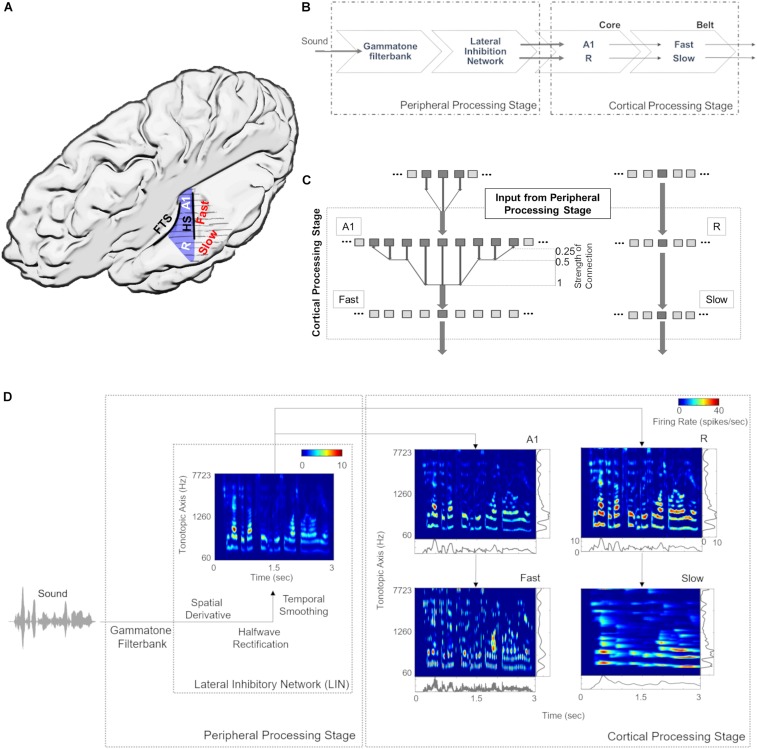
Model design and architecture. **(A)** Anatomical schematic of the modeled areas shown on top view of the left supratemporal plane (with the parietal cortex removed). Heschl’s sulcus (HS) and first transverse sulcus (FTS) are marked to provide anatomical references while Heschl’s Gyrus is highlighted in blue. **(B)** The sound waveform is filtered with a Gammatone filterbank and passed through a Lateral Inhibitory Network (LIN) in the peripheral processing stage, which serves as input to the cortical stage. The neural responses of the simulated core areas (A1, R) are fed forward as input to two simulated belt areas (*Slow* and *Fast*), which differ from each other in their spectral and temporal properties. **(C)** Connections between model stages are shown. The output of Lateral Inhibitory Network (LIN) projects to excitatory units of A1 and R, which in turn project to excitatory units of *Fast* and *Slow*, respectively. While the convergence through A1 to the *Fast* area is high (i.e., many excitatory units of A1 provide input to a single unit of the *Fast* area), convergence through R to the *Slow* area is low (i.e., the units in areas R and *Slow* receive input from only one unit). **(D)** Model output for a sample speech sound is shown at different stages of processing as a spectrogram. The panels at right and bottom of the output of cortical processing stage show mean firing rates across time and tonotopic axis respectively.

Figure 1A provides an anatomical schematic of the modeled cortical areas with approximate locations shown on the left supratemporal plane. Figure 1B illustrates the overall architecture of the model, consisting of a *peripheral* processing stage and a *cortical* processing stage. The *peripheral* processing stage simulates the peripheral auditory processing in two steps. First, the tonotopic response of the cochlea is estimated using a set of band-pass filters (Gammatone filterbank, *N* = 100) (Patterson, 1986; Patterson et al., 1992). The gains of the filters represent the transfer function of the outer and middle ear (4th order Gammatone filterbank implementation by Ma et al., 2007). Following the results from psychoacoustics, the center frequencies of the filters are equally spaced on an ERB_*N*_ number scale and their bandwidth increases with center frequency, so as to have a constant auditory filter bandwidth (Glasberg and Moore, 1990). Thus, bandwidth of the 100 rectangular filters is set as 1 ERB (Equivalent Rectangular Bandwidth, based on psychoacoustic measures; for a review of critical bandwidth as a function of frequency, see Moore, 2003). The filter frequencies are centered from 50 to 8000 Hz, equally spaced with a distance of 0.3 Cams (on the ERB_*N*_ number scale, ERB_*N*_ is the ERB of the auditory filters estimated for young people with normal hearing; Glasberg and Moore, 1990).

Second, the basilar response of the Gammatone filterbank is spectrally sharpened using a Lateral Inhibitory Network (LIN) implemented in three steps by taking a spatial (tonotopic) derivative, half-wave rectification and temporal integration (Chi et al., 2005). The output of extreme filters (i.e., first and last filter) is removed to avoid any boundary effects of filtering, thus reducing the output of the *peripheral* processing stage to 98 units (60–7723 Hz).

For the *cortical* processing stage, the filtered tonotopic cochlear input is processed in two primary auditory *core* areas (A1 and R) and then fed forward to two secondary auditory *belt* areas (*Slow* and *Fast*; Figure 1). These four areas approximate the known architecture of human (Galaburda and Sanides, 1980; Rivier and Clarke, 1997; Wallace et al., 2002) and non-human primates (Hackett et al., 1998; Kaas and Hackett, 2000; Read et al., 2002) AC. Simulated areas primarily differ in their temporal and spectral (spatial) response properties. Specifically, neuronal units in the *Fast* area (approximating caudomedial-caudolateral areas) are characterized by fast temporal dynamics and coarse spectral tuning, whereas units in the *Slow* area (approximating middle lateral-anterolateral areas) are characterized by slow temporal dynamics and fine spectral tuning. It is important to note that these units represent an abstraction at the level of neural population behavior and are not always indicative of single-neuron properties.

In addition, we introduce an interdependence between temporal and spatial (tonotopic) processing within the two *belt* areas, as the variable that determines the temporal dynamics of the responses varies with frequency. Consequently, the units corresponding to lower frequencies in the tonotopic axis respond more slowly than those corresponding to higher frequencies (see Scott et al., 2011; Simpson et al., 2013; Heil and Irvine, 2017). Each simulated area comprises 98 units, which are modeled by excitatory and inhibitory unit pairs. Each of the excitatory core units receives tonotopic input from the corresponding frequency-matched *peripheral* stage. This input only targets the excitatory units of A1 and R. Excitatory responses of A1 and R act as tonotopic input for *Fast* and *Slow* areas, respectively (Figure 1C). The output (excitatory responses) at different stages of the model is shown in Figure 1D.

### The WCCM

Neuronal units of the cortical areas were simulated using the WCCM in MATLAB (The MathWorks, Inc.). The WCCM is a recurrent firing rate model where neural population processes are modeled by the interaction of excitatory and inhibitory responses. The model dynamics are described by Wilson (1999):

(1) $$\text{τ}\frac{\text{d}\,E_{n}\,\left(\text{t}\right)}{\text{dt}}=-E_{n}\left(\text{t}\right)+S_{E}(\sum_{m}w_{{\mathrm{EE}}_{\mathrm{mn}}}E_{n}\left(\text{t}\right)-\sum_{m}w_{{\mathrm{IE}}_{\mathrm{mn}}}I_{n}\left(\text{t}\right)+P_{n}\left(t\right))$$

(2) $$\text{τ}\,\frac{\text{d}\,I_{n}\,\left(\text{t}\right)}{\text{dt}}=-I_{n}\,\left(\text{t}\right)\,+S_{I}\,\,\left(\sum_{m}w_{{\mathrm{EI}}_{\mathrm{mn}}}\,E_{n}\,\left(\text{t}\right)-\sum_{m}w_{{\mathrm{II}}_{\mathrm{mn}}}\,I_{n}\,\left(\text{t}\right)\right)$$

where *E*_*n*_ and *I*_*n*_ are the mean excitatory and inhibitory firing rates at time *t* at tonotopic position *n*, respectively. *P*_*n*_ is the external input to the network and τ is the time constant. The sigmoidal function *S*, which describes the neural activity (Sclar et al., 1990), is defined by the following Naka-Rushton function:

(3) $$S\,\left(P\right)=\frac{{\mathrm{MP}}^{2}}{\theta^{2}+P^{2}}$$

*θ* is the semi-saturation constant and *M* is the maximum spike rate for high-intensity stimulus *P*. The excitatory and inhibitory units are connected in all possible combinations (E–E, E–I, I–E, I–I). The spatial spread of synaptic connectivity between the units *m* and *n* is given by the decaying exponential *w*_*ij*_ (*i*, *j*= E, I) function:

(4) $$w_{{\mathrm{ij}}_{\mathrm{mn}}}=b_{\mathrm{ij}}\,\exp\,\left(\frac{-\left|m-n\right|}{{\text{σ}}_{\mathrm{ij}}}\right)$$

In Equation (4), *b*_*ij*_ is the maximum synaptic strength and σ_*ij*_ is a space constant controlling the spread of activity. The equations were solved using Euler’s method with a time step of 0.0625 ms.

### Parameter Selection and Optimization

Model parameters were selected and optimized based on the following procedure. First, the stability constraints of the model, as derived and implemented by Wilson (1999) were applied. Second, parameters range were chosen so that the model operates in active transient mode, which is appropriate to simulate activity in sensory areas (Wilson and Cowan, 1973). In active transient mode, recurrent excitation triggers the inhibitory response, which in turn reduces the network activity. The balance of excitation and inhibition was achieved by fixing the parameters as described in Table 1 (for the derivation of these parameters see Wilson, 1999). As shown in previous modeling endeavors (Loebel et al., 2007; May et al., 2015), it is crucial to understand the behavior generated through the interaction of various model properties rather than the exact values of the parameters. In our case, we are interested in the interaction of spectral selectivity and temporal dynamics in neural populations constrained by known physiological response properties of the AC. Thus, while most of the parameters were fixed, further tuning was performed to find the combination of spatial spread (σ), connectivity between areas and time constant (τ) such that the areas reflected the general spectral and temporal constraints, as derived from the electrophysiology literature (see following subsections).

**TABLE 1:** Fixed parameters of the model.

Parameters	Values
*M*	100
*θ* inhibition	60
*θ* excitation	80
*b*_*EE*_	1.5
*b*_*EI*_ = *b*_*IE*_	1.3
*b*_*II*_	1.5
*σ_*II*_*	10

**M is the maximum spike rate, θ the is semi-saturation constant. Parameters b_*EE*_*, *b_*II*_ b_*EI*_*, *and b_*IE*_*, *represent the maximum synaptic strength between excitatory units, between inhibitory units, from excitatory to inhibitory units, and vice versa, respectively. All the listed parameter values are same across the four simulated areas.**

#### Spatial Resolution of the Model

Model parameters, spatial spread (σ) and connectivity between areas, were determined by matching the sharpness of the model’s resulting frequency tuning curves (FTCs) with values reported in the literature. FTCs represent the best frequency of auditory cortical neurons as well as their frequency selectivity (i.e., the sharpness of frequency tuning; Schreiner et al., 2000). In primate AC, the sharpness of neuronal FTCs varies from sharp to broad. Quality factor (*Q*) has been used to express the sharpness of the FTCs ($Q=\frac{B\,e\,s\,t\,F\,r\,e\,q\,u\,e\,n\,c\,y}{B\,a\,n\,d\,w\,i\,d\,t\,h}$). The *Q-*values for sharply and broadly tuned auditory cortical neurons have been reported to be around 12 and 3.7, respectively (Bartlett et al., 2011). Also, the core areas have been described as having narrower tuning bandwidths than belt regions (Recanzone et al., 2000). In order to generate narrow FTCs of A1, R, and *Slow* areas and broad FTCs for *Fast* area, we iteratively changed spread of activity within the simulated area (final values are listed in Table 2). When changing the spread of activity (σ) within an area did not affect the *Q* of the area, the connectivity across the areas was manipulated. It should be noted that the projections act as a filter, which is then convolved with the spatial input per unit time. To avoid any boundary effects, symmetric kernel filters (odd number of elements) were used and the central part of the convolution was taken as a result. Final connectivity across regions (i.e., distribution of input units projecting from one area to another) is shown in Figure 1C.

**TABLE 2:** Model parameters across the four simulated areas.

Parameters	Values			
	
	A1	R	*Slow*	*Fast*
τ (ms)	10	20	300–200	3–1
*σ_*EE*_*	40	40	20	200
*σ_*EI*_* = *σ_*IE*_*	160	160	80	300

**For the four simulated areas, the values for varying parameters, time constant τ (reported over the tonotopic axis from low to high best frequencies of the units), spatial spread parameter σ (EE, EI/IE) are listed.**

**FIGURE 2:**
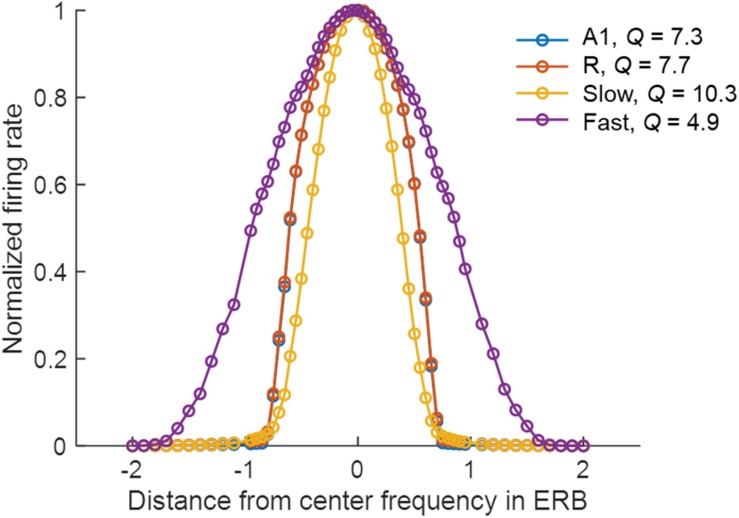
Frequency tuning curves (FTCs) of the unit with best frequency at 4.3 kHz across simulated areas. Areas A1 (blue line) and R (red line) are sharply tuned, with *Q* of 7.3 and 7.7, respectively. The *Slow* area (yellow line) has the sharpest tuning curves with *Q* of 10.3, while *Fast* (purple line) has the broadest tuning with *Q* of 4.9. *Q* is measured as the ratio of the best frequency and the half-maximum bandwidth in Hz.

The narrower tuning in the *Slow* area results from the smaller spread of excitation (σEE, see Table 2), and from the one-to-one projection from R units (Figure 1C). The broader tuning in the *Fast* area is simulated by a many-to-one projection from the Gammatone filterbank to a single unit of A1 (three to one) and from A1 to the *Fast* areas (nine to one). The strength of these connections is shown in Figure 1C. The FTCs across areas are quantified using *Q* at half-maximum bandwidth. The units tuning in the simulated A1 and R areas have mean *Q* = 6.32 (std = 1.43), units in the *Fast* area have mean *Q* = 4 (std = 0.87), while units in the *Slow* have *Q* = 8.35 (std = 2.1). In line with the experimental observations (Kuśmierek and Rauschecker, 2009), the *Q*-values increased with increasing center frequencies, while maintaining the general trend of broad tuning in *Fast* and narrow tuning in *Slow* area. Figure 2 shows FTCs across the four simulated areas for a single unit with best frequency at 4.3 kHz.

#### Temporal Resolution of the Model

Temporal structure represents an important aspect of natural acoustic signals, conveying information about the fine structure and the envelope of the sounds (Giraud and Poeppel, 2012). In several species, a gradient of temporal responses has been observed in AC, with higher stimulus-induced phase locking (synchrony) and lower latencies in area AI compared to adjacent areas (AI vs. AII in cats: Bieser and Müller-Preuss, 1996; Eggermont, 1998; AI vs. R and RT in monkeys: Bendor and Wang, 2008). Correspondingly, model parameters determining the temporal properties of population responses in the simulated areas were adjusted to match such electrophysiological evidence. Table 2 shows the resulting time constant τ for the simulated areas. Note that the values of parameter τ do not represent the latency of the first spike measured for single neurons but affect the response latencies and dynamics at a population level.

##### Temporal latencies

As neurons in core area R have longer latencies than A1 (Bendor and Wang, 2008), we selected a higher value of τ for simulated R than A1. Based on the evidence of the caudomedial field showing similar latencies to A1 (Recanzone et al., 2000; Kuśmierek and Rauschecker, 2014), we adjusted τ of the *Fast* area so that the area is as fast as A1. In contrast, we set τ of the *Slow* area such that this region generates a more integrated temporal response, with the firing rate taking longer to reach the semi-saturation point. These τ values, in combination with the spatial connectivity constraints, cause the simulated belt area to display a spectro-temporal tradeoff. Additionally, in both *Slow* and *Fast* areas τ decreases linearly along the spatial axis (maximum and minimum values are reported in Table 2) with increasing best frequency, following electrophysiological evidence of interaction of the temporal and frequency axis where shorter latencies have been found to be correlated with high best frequencies in macaques (Scott et al., 2011).

##### Temporal synchrony

To further refine parameter τ, next we examined stimulus-driven phase locking of the simulated neural activity. Electrophysiological measurements report synchronization in the neural response to the sound carrier and envelope for a limited range of frequencies, and the upper limit of this phase locking has been found to decrease along the auditory pathway (Joris et al., 2004). At the level of cortex, while the strongest synchronization is reported for modulation rates up to 50 Hz (AM stimuli: Liang et al., 2002, Clicks: Nourski et al., 2013), weaker synchronization to even higher rates (up to 200 Hz) has been observed for a subset of units (Steinschneider et al., 1980; Bieser and Müller-Preuss, 1996; Lu et al., 2001; Nourski et al., 2013). In light of the evidence above, we adjusted τ to mimic this behavior and have strongest temporal synchronization for the low range of modulation rates (up to 50 Hz), with some residual synchronization to higher rates.

### Model Evaluation

The model performance was evaluated in three stages. First, we simulated the electrophysiological coding of AM (for both noise and tone carriers). Second, we evaluated the model’s ability to predict results of human psycho-acoustical tasks, including the determination of amplitude modulation detection threshold functions, tMTFs and perception of missing fundamental. Lastly, we used speech stimuli to investigate the representation of pitch and AM features of a complex sound across the simulated areas. All artificial stimuli (AM noise, AM tones and missing fundamental complex tones) were generated using MATLAB with a sampling rate of 16 kHz and 1 s duration). Speech stimuli were taken from LDC TIMIT database (Garofolo et al., 1993). In all cases, the key readouts of the model were synchronization to stimulus features and firing rates. The pitch estimates matched against model output, where relevant, were computed using the YIN algorithm (de Cheveigné and Kawahara, 2002).

#### Coding of AM Stimuli: Evidence From Electrophysiology

To evaluate the model’s coding of AM, sinusoidally amplitude modulated (sAM) stimuli were used. AM sounds were defined by (1+m⁢sin⁡2⁢π⁢g⁢t)⁢c*⁢a⁢r⁢r⁢i⁢e⁢r, where *m* is the modulation depth, *g* is the modulation rate and *t* is time. The modulation rates were chosen to be 2–9 Hz (linearly spaced), and 10–1000 Hz (logarithmically spaced). Broadband noise was used as carrier to study the response of all units working together while pure tones (500 Hz–3 kHz–5 kHz) were employed to evaluate carrier-specific effects on amplitude modulation coding.

To quantify synchronization of responses to the temporal structure of AM sounds, we employed two measures from the electrophysiology literature (Eggermont, 1991; Joris et al., 2004; Bendor and Wang, 2008): vector strength $\left(\mathrm{VS}=\frac{\mathrm{Strength}\,\mathrm{of}\,\mathrm{Fourier}\,\mathrm{Component}\,\mathrm{at}\,\mathrm{the}\,\mathrm{Modulation}\,\mathrm{Rate}}{\mathrm{Average}\,\mathrm{Firing}\,\mathrm{Rate}}\right)$ (Goldberg and Brown, 1969), and rate modulation transfer function (rMTF), which is the average firing rate as a function of modulation rate. VS was computed for all modulation rates (and three harmonics), for both tone and noise carriers, across the four simulated areas. We considered a simulated area as being synchronized to a modulation rate when VS was greater than 0.1 (this is an arbitrary threshold chosen to compare phase-locking across conditions and areas).

rMTFs were calculated from the average firing rates (i.e., the Fourier component at 0 Hz) and normalized for all areas. For the computation of rMTFs, the modulation depth is fixed at 100% across all AM stimuli. For noise carriers, the computation of the VS and rMTF is based on the mean across all 98 excitatory channels. For the tone carriers, only the channel maximally tuned to the carrier frequency is considered.

#### Simulating Psychoacoustical Observations

The model was tested using three paradigms approximating human psychoacoustic studies. The first two experiments simulated temporal modulation transfer functions (tMTFs: quantifying the modulation depth required to detect different modulation rates) for broadband noise (Bacon and Viemeister, 1985) and tones (Kohlrausch et al., 2000). The third experiment simulated pitch identification with missing fundamental stimuli (Houtsma and Smurzynski, 1990).

For the simulated tMTFs, AM sounds with incremental modulation depths (from 1 to 100%) were presented to the model and the oscillations in the model’s output were measured. In the psychoacoustic measurements, the lowest modulation depth at which subjects can detect the modulation is considered the detection threshold. In the model, using synchronization as output measure, the lowest value of modulation depth at which the output is synchronized to the modulation rate (i.e., the strongest Fourier component was at the modulation rate) is considered as the detection threshold for that AM rate. This procedure was repeated for all the modulation rates and, for all simulated areas. For noise carriers, the mean across the excitatory units across each area is analyzed and compared to data collected by Bacon and Viemeister (1985). The model response was simulated for modulation rates at 2–9 Hz (linearly spaced), and 10–1000 Hz (logarithmically spaced).

For AM tones, the analysis of the waveform shows spectral energy at the carrier frequency and at the carrier frequency ± modulation rate. These accompanying frequency components are called “spectral sidebands” of the carrier frequency. If the modulation rate is high enough, these sidebands activate distinctively different auditory channels than the carrier frequency and can be detected audibly apart from the carrier frequency. Thus, for the tone carriers (1 and 5 kHz) the active part of the population (comprising the best frequency channel and spectral sidebands) was used to compute tMTFs based on temporal synchronization to the modulation rate (temporal code) and detection of sidebands (spatial code). As before, for the temporal code, the lowest value of modulation depth at which the output is synchronized to the modulation rate (i.e., the strongest Fourier component was at the modulation rate) is considered as the detection threshold for that AM rate. For the spatial code, the modulation depth at which the side-band amplitude (mean firing rate over time) is at least 5, 10, 15, or 20% of the peak firing rate (firing rate of the channel with CF closest to carrier frequency) are calculated. The best (lowest) value of modulation depth is chosen from both coding mechanisms. The combination of these coding mechanisms is then compared to tMTFs (at 30 dB loudness) reported by Kohlrausch et al. (2000). The modulation rates tested were 10–1600 Hz (logarithmically spaced).

Pitch of missing fundamental complex tones has been shown to be coded by temporal and spatial codes, depending on the order of harmonics and frequency of missing fundamental (Bendor et al., 2012). Here we replicated this finding by simulating the model response to complex tones with low order (2–10) and high order harmonics (11–20) and varying missing fundamental frequency from 50 to 800 Hz. The synchronization to the missing F_0_, measured in VS, is computed from the mean responses over time in each of the four simulated areas. Furthermore, to evaluate the role of synchronization in pitch perception, we simulated model responses to complex tones with unresolved harmonics of a missing fundamental frequency by approximating a pitch identification experiment by Houtsma and Smurzynski (1990). The missing fundamental tone complexes vary in two aspects: the number of harmonic components (2–11) and the lowest harmonic component (10 and 16) while the fundamental frequency (F_0_) is fixed at 200 Hz. For each combination of lowest harmonic component and number of components in the harmonic complex, we computed the synchronization to the F_0_ (in VS) and mean firing rates for all four regions.

#### Model Responses to Speech

Model responses to the speech stimuli were analyzed in two stages. The speech stimuli (630 sentences, all spoken by different speakers; mean duration 3.4 s) were randomly selected from the LDC TIMIT database (Garofolo et al., 1993). To study how key temporal features of speech waveforms are represented in the modeled areas, we compared the temporal modulations in the output of all four simulated areas to the temporal modulations of the input signals. To this end, we computed the input-output magnitude spectrum coherence (*mscohere* in MATLAB with a 2048 point symmetric hamming window and overlap of 1500 samples) between the input speech signal (after LIN) and the output of all four areas. The coherence values are then scaled across the four areas using the mean spatial activity along the tonotopic axis (i.e., the mean firing rate over time for all sounds). To highlight the difference in spectrum coherence between the spectro-temporal processing streams in the model, the difference between the scaled input-output coherence is computed to compare the two *core* (R–A1) regions to each other and the two *belt* areas (*Slow*–*Fast*).

## Results

### Coding of AM Stimuli

We investigated the model’s AM coding using both broadband noise and tone carriers. By using broadband noise as carrier, we simulated general responses for each of the four areas, and then used pure tone carriers to study the dependence of the synchronization and rate coding on the tonotopic location (i.e., the best frequency of the units).

#### Sinusoidal AM Noise

**FIGURE 3:**
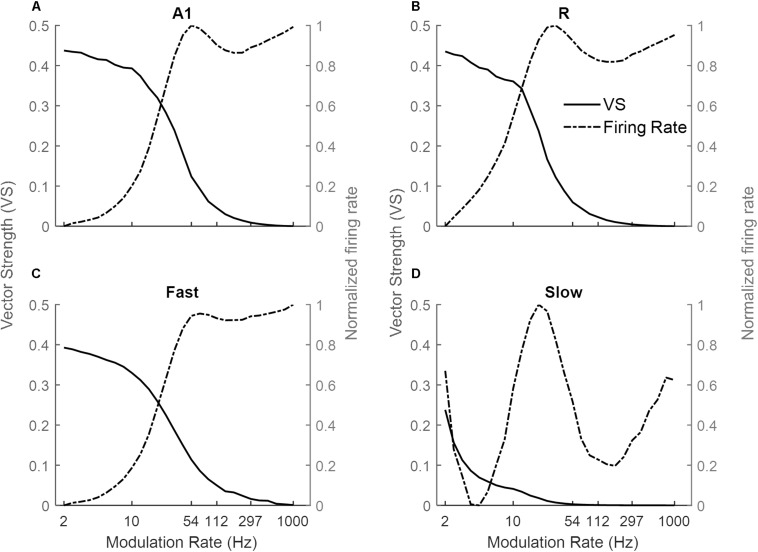
Model responses to sAM noise across simulated areas. A dual coding mechanism for modulation rates, i.e., temporal (measured as Vector Strength, VS, solid lines) and rate codes (quantified as the rate Modulation Transfer Functions, rMTFs, dash-dotted lines), are shown for A1, R, *Fast*, and *Slow* areas in **(A–D)** respectively. In A1, R, and *Fast* areas, the synchronization decreases for higher modulation rates and is complimented by increasing firing rate. While very little synchronization is observed in the *Slow* area, the respective rMTF shows an interesting band-pass profile.

Figure 3 shows the response of the four simulated cortical areas (A1, R, *Fast*, and *Slow*) as a function of the modulation rate of sinusoidally amplitude modulated (sAM) noise. We analyzed the mean response of all units for each area. Across regions, the response synchronization (measured as VS) decreases with increasing modulation rate (solid lines in Figures 3A–D for A1, R, *Fast*, and *Slow* areas respectively). The decrease in synchronization is observed to be rapid above an area-specific modulation rate (8 Hz for A1, R and *Fast* areas, 2 Hz for *Slow*). Taking the lower limit for synchronization as VS = 0.1, the highest AM rate to which the areas synchronize is 54 Hz in A1, 33 Hz in R, 4 Hz in *Slow* and 54 Hz in *Fast*. Overall, the observed responses to modulation rates show a low-pass filter profile.

Instead, the firing rate [rate Modulation Transfer Functions (rMTFs), dash-dotted lines] shows different behavior across the four areas in response to AM noise. For A1, R and *Fast* areas (Figures 3A,C respectively), the firing rate does not change for lower modulation rates (until 10 Hz for A1 and *Fast*, until 6 Hz for R) and then rapidly increases until a maximum limit (54 Hz for A1, R and *Fast*) and does not further change in response to higher modulation rates. In contrast, the firing rate in the *Slow* area (Figure 3D) shows a band-pass profile between 6 and 100 Hz, peaking at ∼20 Hz.

#### Sinusoidal AM Tones

**FIGURE 4:**
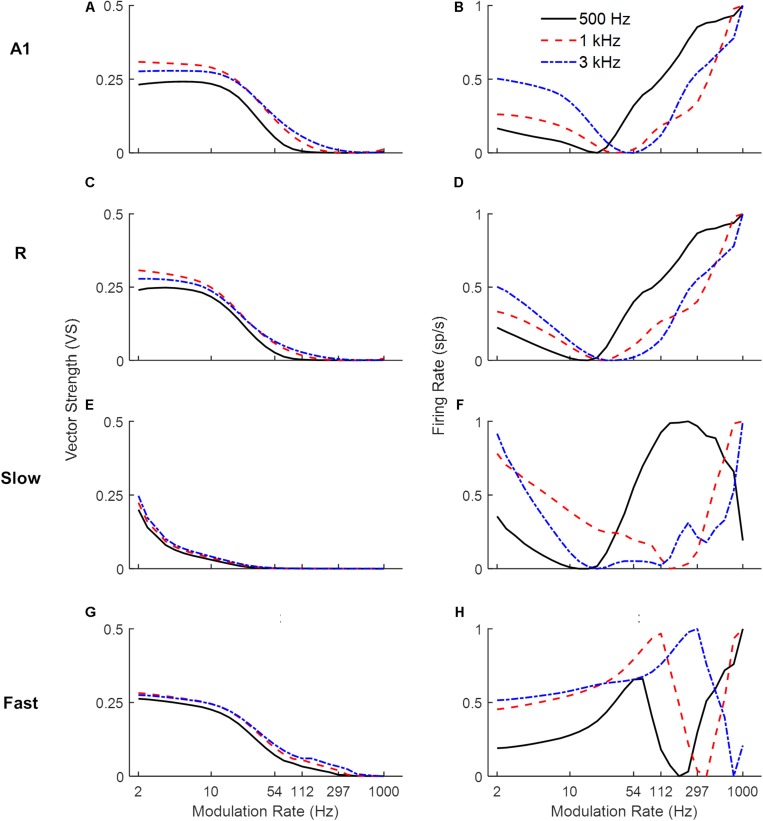
Model responses to sAM tones across simulated areas. A dual coding mechanism for modulation rates, i.e., temporal (measured as Vector Strength, VS, left panels) and rate codes (quantified as the rate Modulation Transfer Functions, rMTFs, right panels), are shown for A1, R, *Fast* and *Slow* areas in respective panels (A1: **A,B**, R: **C,D**, *Slow*: **E,F**, *Fast*: **G,H**). For the three different carriers, synchronization to higher modulation rates is observed with increasing carrier frequencies across areas **(A,C,E,G)**. Rate coding, however, shows more varied profiles with different carriers **(B,D,F,H)**.

Next, we explored the frequency dependence of AM processing. As the use of broadband noise as a carrier provides no information about the temporal properties of different frequency channels along the tonotopic axis, we simulated model responses to AM pure tone carriers. Figure 4 shows response synchronization (VS, left column) and firing rate (rMTFs, right column) across cortical areas as a function of AM rate, separately for units best responding to a low (solid lines), middle (dashed lines), and high (dash-dotted lines) frequency pure tone carriers (500, 1k and 3k Hz respectively). For each area, the responses in the model’s frequency channel matching the tone carrier are shown. The synchronization shows a low-pass filter profile consistently for all three carriers. With increasing carrier frequency, the A1, R, and *Slow* areas (Figures 4A,C,E) are synchronized (VS cut-off at 0.1) to higher modulation rates (A1: 33 Hz for 500 Hz, 54 Hz for 1 kHz and 3 kHz, R: 26 Hz for 500 Hz, 33 Hz for 1 kHz and 3 kHz, *Slow*: 3 Hz for 500 Hz, 4 Hz for 1 and 3 kHz). This behavior is consequence of the relationship between the temporal and spatial axis (a property of the model), with temporal latencies reducing with increasing center frequencies of the units allowing phase-locking to higher modulation. The *Fast* area (Figure 4G) shows a similar cutoff for all carriers at 54 Hz. The rMTFs (Figures 4B,D,F,H for areas A1, R, *Slow*, and *Fast* respectively), however, show more complex and varied behavior for different carriers (including monotonically increasing, band-pass, and band-stop behavior). This behavior is in line with rMTFs from electrophysiological studies, where instead of singular behavior (like low-pass filter profile reported for tMTFs), rMTFs show variety of response profiles (Schreiner and Urbas, 1988; Bieser and Müller-Preuss, 1996; Liang et al., 2002; Bendor and Wang, 2008).

### Simulating Psychoacoustic Observations

Next, the model was tested using three experimental paradigms similar to those employed in human behavioral studies. The first two experiments tested the temporal modulation transfer functions (tMTFs characterizing the modulation depth required to detect different modulation rates) for broadband noise (Bacon and Viemeister, 1985) and tones (Kohlrausch et al., 2000). The third experiment examined the effects of the number of harmonics in pitch identification with missing fundamental stimuli (Houtsma and Smurzynski, 1990).

#### Temporal Modulation Transfer Functions for Broadband White Noise

**FIGURE 5:**
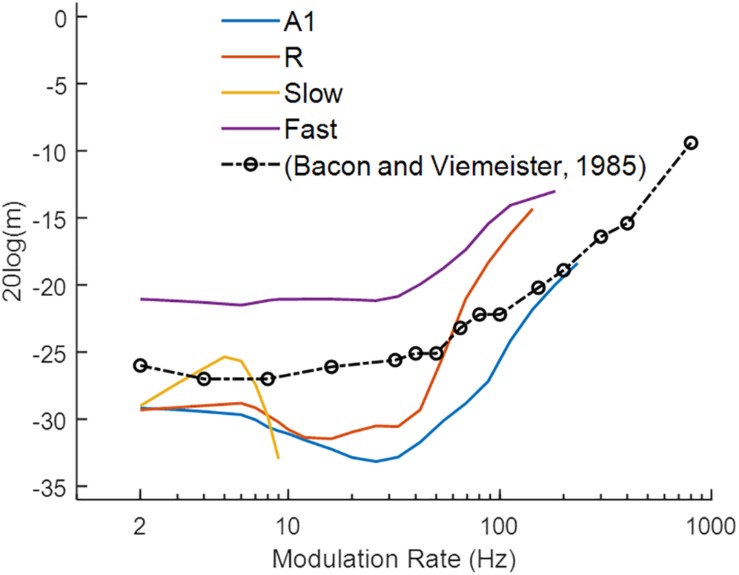
Modulation detection with sAM noise. The temporal Modulation Transfer Functions (tMTFs), illustrating the minimum depth required to detect the amplitude modulation in sAM noise, are shown for the four model areas (in colored lines) and for a psychoacoustic study (black line and circles; adapted from Bacon and Viemeister, 1985). Lower values depict higher sensitivity to modulation rate. Modulation depth, m (dB) of the signal is plotted on y-axis.

Similar to the behavioral task of Bacon and Viemeister (1985), we measured responses of the model to AM sounds with variable modulation depth and recorded the minimum modulation depth where the output signal was synchronized to the modulation rate (i.e., the strongest Fourier component was at the modulation rate) of the AM noise. Figure 5 illustrates the simulation results (solid colored lines), along with human psychoacoustic data (dash-dotted black lines with circles, adapted from Bacon and Viemeister, 1985). Lower values depict higher sensitivity to the modulation rates. A1 and R show lower thresholds for slower than for faster modulation rates. In the *Fast* area, the detection profile is similar to A1 and R, but the minimum detection depth is higher than in the other areas. The broad tuning of the *Fast* area reduces the precision of the temporal structure of the input signal. Thus, the *Fast* area performs worse than the other areas across modulation rates. In the *Slow* area, modulation detection is observed to be limited to rates below 10 Hz. Thus, the *core* areas outperformed the *belt* areas in the detection of amplitude modulations. The modulation depth detection profile of the *core* areas resembles the results from human psychophysics suggesting that primary auditory cortical processing may underlie tMTFs reported in psychophysics. In comparison with synchronization, rate coding is difficult to quantify as observed before with varying response profiles for rMTFs along the frequency axis (Figures 4F,H). The difference between our simulations and psychophysical findings at faster rates may be explained by the fact that our simulations only considered coding through response synchronization and ignored the contribution of rate coding contributing to the detection of higher modulation rates.

#### Temporal Modulation Transfer Functions of Sinusoidal Carriers

**FIGURE 6:**
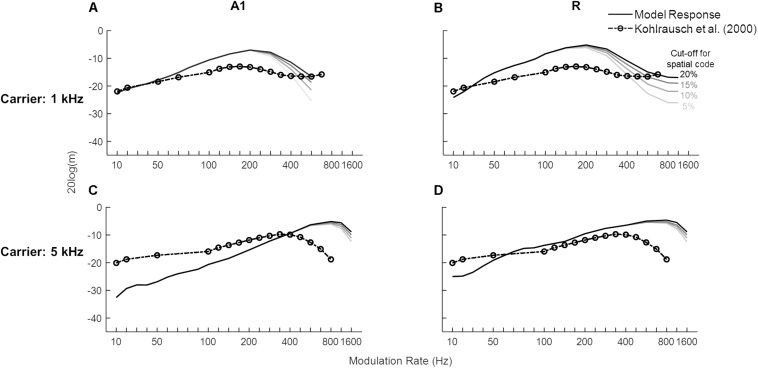
Modulation detection with sAM tones. The solid lines show the temporal Modulation Transfer Functions (tMTFs), illustrating the minimum depth required to detect the amplitude modulation in sAM tones (1 kHz in top panels, 5 kHz in bottom panels), are shown for the two *core* areas (A1 in **A,C**, R in **B,D**). The model output is a combination of temporal and spatial codes for modulation detection. Variation in the spatial code is shown at four different cut-off values, represented by the solid lines in different gray-scales. Data from a psychoacoustic study are shown in dash-dotted lines with circles (adapted from Kohlrausch et al., 2000). Lower values depict higher sensitivity to modulation rate. Modulation depth, m (dB) of the signal is plotted on y-axis.

We then investigated the model’s detection threshold function of sAM tones. Psychoacoustic studies have shown that human performance does not change across the lower modulation rates, becomes worse for a small range and then improves after the side-bands introduced by the modulation become detectable (Sek and Moore, 1995; Kohlrausch et al., 2000; Moore and Glasberg, 2001; Simpson et al., 2013). We obtained model responses to sAM tones as a combination of temporal and spatial codes. To characterize an area’s modulation detection threshold represented by temporal code, the lowest modulation depth at which the best frequency unit or the spectral sideband synchronized to the modulation rate was chosen. Additionally, the spatial code was quantified by detection of spectral sideband. Figure 6 shows the lowest modulation depth for which A1 (solid lines in Figures 6A,C) and R (solid lines Figures 6B,D) code modulation rates of sAM tones and the psychoacoustic data for 1 and 5 kHz sinusoidal carriers at 30 dB (dash-dotted lines with circles, Kohlrausch et al., 2000). The initial increase in depth values indicates the contribution of temporal coding of the modulation rates that gets worse with higher modulation rates. With increasing modulation rates, however, the spectral sidebands dissociate from the carrier channel and the contribution of spectral coding is observed. The modulation depths at which the sideband amplitude (mean firing rate over time) is detectable (multiple threshold cut-offs are shown where sideband activity is 5, 10, 15, and 20% of the firing rate of the channel with CF closest to carrier frequency) are also shown in Figure 6. No synchronization is observed in the *Slow* and *Fast* areas. Overall, model results show a clear frequency dependence as detection of higher rates was observed for the higher carrier (maximum for A1: 500 Hz for 1 kHz carrier, 1.2 kHz for 5 kHz carrier; R: 1.2 kHz for 1 kHz carrier, 1.6 kHz for 5 kHz carrier). The modulation detection by the model slightly worsened with increasing modulation rate but improved (lower m values) as the sidebands introduced by the modulation became detectable (after 100 Hz for the 1 kHz carrier in A1 and R, after 400 Hz for 5 kHz carrier in A1). This improvement of AM detection threshold for high AM rates is in accordance with human psychophysics, where observations show a decrease in performance with increasing modulation rates is followed by a performance increase accompanied with side-band detection (Sek and Moore, 1995; Kohlrausch et al., 2000; Moore and Glasberg, 2001; Simpson et al., 2013). Additionally, matching the model results, human psychophysics show improved performance (i.e., detection of higher rates) with increasing carrier frequencies.

#### Pitch of Missing Fundamental Sounds

**FIGURE 7:**
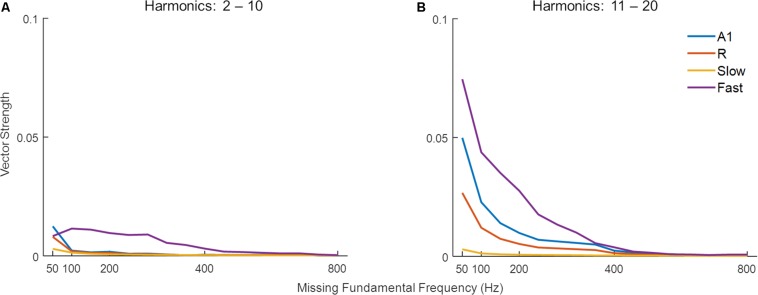
Synchronization to missing fundamental frequency across harmonic order. The model performance in detecting missing fundamental of complex tones (measured as vector strength) with **(A)** low-order harmonics, and **(B)** high-order harmonics. Simulated responses in the four areas are shown in different colors.

Missing fundamental sounds are harmonic complexes that, despite lacking energy at the fundamental frequency (F_0_), induce the percept of a pitch corresponding to F_0_ (Yost, 2010; Oxenham, 2012). If the harmonic components in the missing fundamental sound are resolved (i.e., each component produces a response on the basilar membrane that is distinct from that of neighboring harmonic components), the pitch information can be extracted through a spectral (spatial) mechanism, or a temporal mechanism if harmonics are unresolved, or a combination of the two (Yost, 2009). Bendor et al. (2012) have shown that low F_0_ sounds with higher-order harmonics are primarily represented by temporal mechanisms. Thus, we tested the effect of harmonic order on the detection of missing F_0_ through temporal synchrony across simulated areas. Figure 7 shows synchronization (temporal code, measured as VS) to missing F_0_ of complex tones with lower-order and higher-order harmonics in panels A and B respectively. Stronger synchronization is observed for higher-order harmonics compared to lower-order harmonics for lower missing F_0_ complex tones in A1, R, and *Fast* areas. The effect is most pronounced in the *Fast* area. However, the synchronization drops with increasing missing F_0_, and very little to none synchronization is observed after 400 Hz irrespective of the order of harmonics in the complex tone.

**FIGURE 8:**
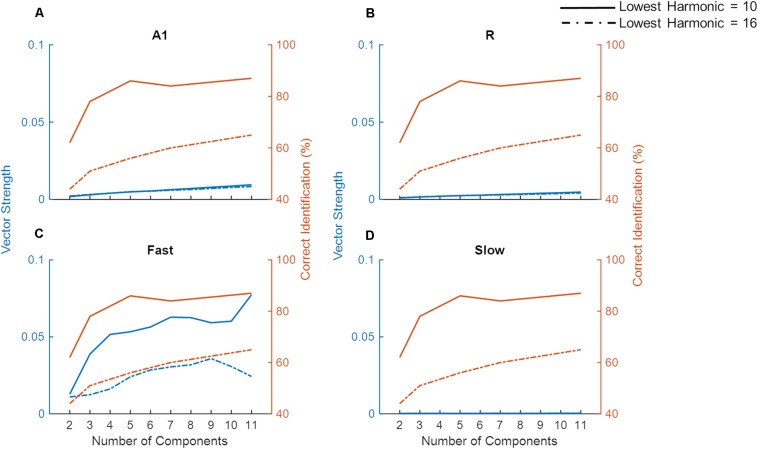
Model performance on a missing fundamental task. The model performance in detecting missing fundamental of complex tones (synchronization to missing fundamental frequency at 200 Hz, measured as Vector Strength) is shown for areas A1, R, *Fast*, and *Slow* (blue lines in **A–D**, respectively). Human behavioral data on pitch identification (%) task (Houtsma and Smurzynski, 1990) is plotted in orange lines. Solid lines show complex tones with lowest harmonic at 10 while the dash-dotted lines show the lowest harmonic component at 16.

For low pitch missing fundamental sounds, psychophysics experiments employing sounds with unresolved harmonics have shown that humans are better at identifying a missing fundamental pitch when the sound consisted of lower (lowest harmonic = 10) compared to higher unresolved harmonics (lowest harmonic = 16), yet the performance reaches a plateau as more harmonics components are included for the sound consisting of lower but not higher-order harmonics (Houtsma and Smurzynski, 1990). To evaluate whether temporal mechanisms play a role in these findings we simulated a pitch identification experiment (Houtsma and Smurzynski, 1990) and explored the effects of the number of harmonic components and lowest order harmonic in the missing fundamental complex tone on the model’s behavior. As already established, simulated populations could only successfully synchronize to lower missing F_0_ (Figure 7), thus the task employed complex tones with low missing F_0_ (200 Hz). Figure 8 shows the model’s synchronization (VS) to the missing F_0_ (200 Hz and the first three harmonics) across the simulated regions (in blue lines), along with the results from the psychophysics experiment (in red lines, data adapted from Houtsma and Smurzynski, 1990).

While we did not observe any differences due to harmonic order in VS measured in A1, R, and *Slow* areas (Figures 8A,B,D), the *Fast* area (Figure 8C) showed clear dissociation in synchronization code when the lowest order harmonic changed from 10 to 16. That is, the synchronization to the missing F_0_ in the *Fast* area was stronger when the lowest order harmonic was 10. Additionally, for both complex tones, the performance of the *Fast* area improved with an increasing number of components. The improvement in synchronization was rapid when the number of components changed from 2 to 4 for the lowest order harmonic at 10. These observations are in line with the pitch identification data shown in the red lines. Thus, neural response properties similar to those of the *Fast* area are optimized to temporally detect the F_0_ from missing fundamental sounds, and responses in the *Fast* area follow human behavior.

Unlike synchronization, the simulated firing rate (Supplementary Figure S1) did not show a pattern that matched the behavioral data. Specifically, the simulated firing rate increased monotonically as a function of the number of components in the complex tone, irrespective of the lowest order harmonic.

### Model Responses to Speech

**FIGURE 9:**
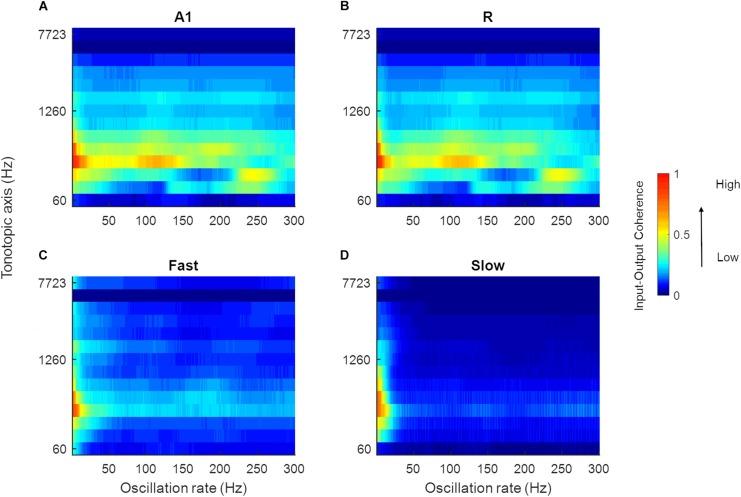
Mean magnitude spectrum coherence between speech sounds and model output. The coherence values in A1, R, *Fast*, and *Slow* areas are shown in **(A–D)**, respectively (scaled by the normalized mean spatial response of the model to 630 speech sounds). All areas show high coherence with the slow oscillations present in the input signal (indicated by red and yellow colors).

Speech signals encode information about intonation, syllables, and phonemes through different modulation rates. We explored the processing of speech sounds across simulated cortical areas to study the importance of simple spectro-temporal cortical properties, as reported by electrophysiology and represented by the model, in coding these temporal features of speech. To this end, we analyzed model output in response to 630 speech stimuli by computing the magnitude spectrum coherence between these sounds (the output of the LIN stage) and the simulated model responses for each of the four areas. Figure 9 shows the normalized coherence plots (scaled by the normalized time-averaged activity). In all regions, we observed model synchronization to slow changes in the stimuli (<20 Hz).

**FIGURE 10:**
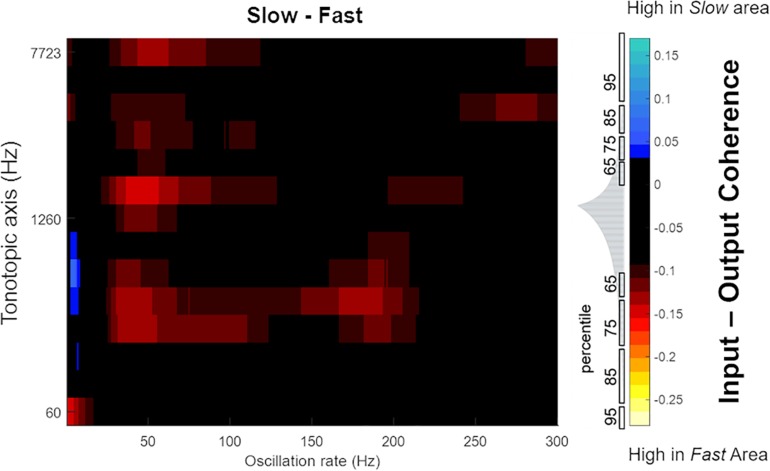
Mean difference in magnitude spectrum coherence between belt regions. The *Slow* area showed higher coherence with slow oscillations in speech (4–8 Hz, indicated by blue colors). Instead, the *Fast* area showed greater coherence to faster oscillations of speech (30–70 Hz, around 100 and 150–200 Hz, indicated by the warmer colors). The distribution of difference in magnitude spectrum coherence between *Slow* and *Fast* area for all 630 sounds is shown in gray, adjacent to the color bar, with percentiles marked to indicate the statistical significance.

Next, in order to highlight differences in the temporal response properties between regions, we computed difference plots for the simulated core and belt areas. While we observed no differences in coding of temporal features between A1 and R, Figure 10 shows that differences are present in the *belt* stream (comparing the coding of temporal features in the *Fast* to those in the *Slow* area). The difference between the coherence (*Slow*–*Fast*) across 630 stimuli (mean: -0.0332, SEM: 0.0041) was used to compute the data distribution in four percentiles (65, 75, 85, and 95%). These percentiles are shown along the color bar in Figure 10 (with the distribution) to provide a threshold for the significance to the difference between input-output coherence of the *Slow* and *Fast* area. Shades of blue show stronger input-output coherence in the *Slow* area, while the warmer colors indicate stronger input-output coherence in the *Fast* stream. The *Slow* area represents the slower changes (4–8 Hz) in the speech envelope better than the *Fast* area. The *Fast* area, on the other hand, highlights faster changes in the temporal structure of speech in two frequency ranges (30–70 Hz, and around 100–200 Hz).

We hypothesized that the higher of these two frequency ranges (100–200 Hz) may reflect the presence of temporal pitch information in the *Fast* area. The temporal code for pitch in the simulated areas was estimated by computing short-time the Fourier Transform (window length: 300 ms, overlap: 200 ms) over length of the signal. The resulting power spectral density estimates showed temporal synchronization to the frequencies approximating the pitch in A1, R and *Fast* areas over time. For the purpose of comparison across simulated areas, the pitch estimates and contour obtained for voiced portions of the sounds (using the YIN algorithm) were correlated with the oscillatory activity of individual simulated areas for all 630 speech stimuli. Mean correlation values were A1: 0.46 (SEM: 0.02), R: 0.47 (SEM: 0.02), *Slow*: -0.14 (SEM 0.01), *Fast*: 0.59 (SEM 0.01), and showed that the *Fast* area best represented the pitch information through synchronization to instantaneous F_0_.

**FIGURE 11:**
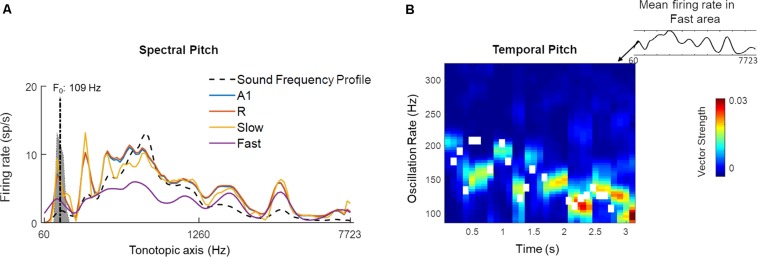
A dual code for pitch estimation. For a sample sound, **(A)** Mean firing rate of all units in the four simulated areas (A1, R, *Slow*, and *Fast*, colored lines) is shown. Sound frequency profile (scaled) is plotted in black dashed line for reference. The gray highlighted portion of the plot indicates estimates of pitch by YIN algorithm (distribution over time, with best estimate of F_0_ plotted with dash-dotted line; de Cheveigné and Kawahara, 2002). A spectral code is observed in model outputs with firing rate peaks overlapping with YIN estimates. **(B)** Temporal code for pitch is observed as weak synchronization to pitch contour in oscillatory activity (measured as Vector Strength) of the *Fast* area unit corresponding to spectral peak corresponding to best pitch estimate by YIN algorithm. The pitch contour estimates over time computed by YIN algorithm are depicted by white boxes. The correlation between YIN estimates and the temporal profile of the *Fast* area is 0.74.

Figure 11 highlights the presence of a dual mechanism for coding pitch, as pitch information is present in both spectral (i.e., spatially, by different units) and temporal (by different oscillatory activity) model responses for a sample sound (male speaker, sentence duration 3.26 s; selected from LDC TIMIT database; Garofolo et al., 1993). In Figure 11A, the time-averaged response to the speech sentence across the tonotopically-organized channels in the four simulated areas is shown. In all the areas, a peak in the response profile can be observed in those frequency channels that matched the F_0_ of the speaker (best estimate computed using YIN algorithm: 109 Hz). This spectral (i.e., spatial) representation of the speech signal’s pitch is strongest in the *Slow* area and weakest in the *Fast* area. A1 and R show similar profiles with respect to each other. Contour tracking of pitch in the *Fast* area with the sample sound (correlation 0.74) is shown in Figure 11B (pitch contour of the speech signal measured by YIN algorithm is shown as the white boxes). The simulated *belt* regions show functional specialization to represent pitch spectrally (in the *Slow* area) and temporally (in the *Fast* area) in parallel streams.

Overall, the model responses to speech sounds highlight the presence of a distributed code for representing different temporal features of speech signals at the level of *belt* regions, but not for the *core* regions. Each *belt* area showed a functionally relevant specialization, as the temporal features highlighted by *Slow* and *Fast* areas are key structures of speech signals.

## Discussion

In this study, we presented a computational model of the AC that consists of information processing streams optimized for processing either fine-grained temporal or spectral information. The model is employed to investigate the contribution of the different cortical streams in the representation and processing of basic acoustic features (i.e., temporal modulation, pitch) in the context of artificial and natural (speech) stimuli.

We started by simulating responses to artificial AM sounds. Electrophysiological studies have characterized AM coding by a dual mechanism of temporal (synchronization) and rate coding (Joris et al., 2004). In comparison with the phase-locking in the auditory nerve (reported up to 1.5–8 kHz in humans; Verschooten et al., 2019), the synchronization code has been measured to be comparatively diminished at the level of the cortex for human and non-human primates. The preferred AM rates have been reported as ranging from 1 to 50 Hz in monkeys (Steinschneider et al., 1980; Bieser and Müller-Preuss, 1996; Lu et al., 2001), despite neurons have been shown to synchronize as high as 200 Hz in monkeys (Steinschneider et al., 1980) and similar weak synchronization could be detected in humans with electrocorticography (Nourski et al., 2013). In agreement with these electrophysiology studies, our model exhibited a dual coding mechanism. While the contribution of a temporal code (synchronization) was strong up to a maximum of 50 Hz, synchronizations became weaker for higher modulation rates and were complemented with a rate code mechanism.

Furthermore, in electrophysiology, the maximum AM rate for which a temporal code is present has been reported to differ across fields of the AC (Liang et al., 2002). Caudal fields (i.e., regions belonging to the dorsal processing stream) are reported to be as fast as or even faster than the primary AC and synchronize with the stimulus envelope up to high AM rates. Instead the rostral field (i.e., part of the ventral processing stream) does not show a temporal code for AM sounds but instead codes AM with changes in firing rate (i.e., a rate code) (Bieser and Müller-Preuss, 1996). In the simulated responses, the relative contribution of the temporal and rate coding mechanisms also varied across the simulated cortical areas, depending upon the areas’ temporal and spectral processing properties. While the temporal code displayed a low-pass filter profile, the shape of the rate code varied from low-pass to band-pass and band-stop patterns. Evidence for such variation in rate coding pattern has been reported in electrophysiological studies as well with sAM stimuli (Schreiner and Urbas, 1988; Bieser and Müller-Preuss, 1996; Liang et al., 2002; Bendor and Wang, 2008). In our model, this observation was highlighted when the firing rate was examined within carrier-matched frequency channels. The interaction of spectral and temporal response properties underlies these observations.

In order to assess the relationship between neural population activity (i.e., synchronization and firing rate) with human behavior, we next used the model to simulate psychoacoustic experiments. We were able to successfully predict psychoacoustically-determined modulation detection thresholds (i.e., modulation detection transfer functions, tMTFs) for AM noise and tones (Bacon and Viemeister, 1985; Kohlrausch et al., 2000). The model suggested a role for auditory *core* areas, rather than *belt* areas, in coding modulation detection with simple AM stimuli. The tMTF for AM noise was replicated by computing temporal synchronization. However, for AM tones, we observed the best prediction of the psychoacoustical tMTF by using a combination of synchronization and spatial (sideband detection) code. Additionally, we observed that compared to low-frequency carriers, high carriers allowed modulation detection up to faster rates. This replicated psychoacoustic observations of detection up to faster modulation rates with a higher carrier frequency (Sek and Moore, 1995; Kohlrausch et al., 2000; Moore and Glasberg, 2001; Simpson et al., 2013). Our simulations indicate that these frequency-specific responses, which arise at the periphery, are inherited by the cortex, especially in the *core* areas.

We further evaluated the contribution of temporal coding mechanisms to psycho-acoustical phenomena. While current views on pitch perception suggest that the role of synchronization is limited to auditory periphery and cortex might use information from individual harmonics (Plack et al., 2014), there is evidence of temporal cues being used especially for unresolved harmonics for low pitch sounds (Bendor et al., 2012). The model successfully decoded the low frequency missing fundamentals of complex tones and showed dependence of strength of synchronization on the order of harmonics. By simulating a psychoacoustic task employing missing fundamental complex tones with varying unresolved harmonics, we further investigated the role of synchronization and its dependence on number and order of harmonics. The model output matched the previously reported human behavior performance through synchronization in the simulated neural responses, but not by a rate coding mechanism. That is, we could successfully replicate three key findings from Houtsma and Smurzynski (1990). First, the synchronization to the missing F_0_ was stronger for the lower compared to higher-order harmonic sounds and second, it improved with an increasing number of components of complex tone. Third, only for the lower order harmonic sounds, the improvement in model performance was sharp when the number of components was increased from two to four and displayed a plateau when further components were added. Interestingly, the match between psychoacoustics and the model output was limited to the *Fast* area, suggesting a role for this fine-grained temporal processing stream in the extraction of the pitch using temporal cues. Additionally, using speech sounds, we further observed a strong spatial (spectral) pitch correlate (observed in all areas, strongest in *Slow* area) along with weaker oscillations tracking pitch contour (only in *Fast* area). However, the spatial code is not observable in model output for pitch with missing fundamental complex tones and suggests need for a more complex network to effectively detect pitch just from harmonic information in space. Moreover, the temporal code for pitch can benefit from feedback connectivity (Balaguer-Ballester et al., 2009) while precise interspike intervals can shed light on phase sensitivity of pitch perception (Huang and Rinzel, 2016). Thus, future model modifications can move from general (current) to more specific hypotheses of auditory processing.

Coding of pitch in the AC has been extensively investigated with fMRI, resulting in somewhat conflicting findings. While some studies pointed to lateral Heschl’s Gyrus (HG) as a pitch center (Griffiths and Hall, 2012; Norman-Haignere et al., 2013; De Angelis et al., 2018), other studies showed that pitch-evoking sounds produced the strongest response in human planum temporale (PT) (Hall and Plack, 2009; Garcia et al., 2010). This disagreement may be due to differences between studies in experimental methods and stimuli. Our computational model provides an opportunity to merge these fMRI-based findings, as it allows for the efficient and extensive testing of model responses to a broad range of sounds. Based on the sounds we tested, observations of a pitch center in PT, part of the *Fast* stream, may be dominated by temporal pitch. Instead, human fMRI studies reporting a pitch area in lateral HG (Griffiths and Hall, 2012; Norman-Haignere et al., 2013; De Angelis et al., 2018), which is part of the *Slow* stream), maybe reflecting the spectral rather than the temporal processing of pitch. Our simulations suggest a functional relevance for temporal representations albeit through weak synchronization. These predictions are in line with evidence of synchronization in the AC contributing to the percept of pitch (up to 100 Hz) observed with MEG (Coffey et al., 2016) and require future studies with both high spectral and temporal precision data from the AC.

The distributed coding pattern shown by the different regions (i.e., coding of modulation detection thresholds by the *core* regions, coding of temporal pitch by the *Fast* area and spectral acuity by the *Slow* area of the *belt* stream) reflected a hierarchical processing scheme based on varying spectro-temporal properties of the neural populations. We then applied this modeling framework to the analysis of (continuous) speech with the aim of exploring the influence of basic neural processing properties on the representation and coding of speech. All modeled areas represented the slow oscillations present in speech (<20 Hz). In the belt areas, an additional distributed coding of temporal information was observed. That is, the optimization for coding slow temporal changes with high spectral precision in the *Slow* stream resulted in the coding of temporal oscillations in the lower 4–8 Hz frequency range. Processing properties similar to those of the *Slow* stream may thus be suited for coding spectral pitch and prosody in speech signals. Instead, optimization for processing fast temporal changes with low spectral precision in the *Fast* stream resulted in coding of temporal oscillations in the higher 30–70 and 100–200 Hz frequency ranges. Processing properties similar to those of the *Fast* stream may therefore instead be optimal for coding phonemes (consonants), and temporal pitch. In sum, we showed that the hierarchical temporal structure of speech may be reflected in parallel and through distributed mechanisms by the modeled areas, especially by simulated belt areas. This is in line with the idea that the temporal response properties of auditory fields contribute to distinct functional pathways (Jasmin et al., 2019).

The “division of labor” observed between the simulated processing streams provides predictions regarding cortical speech processing mechanisms. Specifically, the slowest oscillations, representing the speech envelope, were coded in parallel across regions with different processing properties and may serve to time stamp the traces of different speech aspects belonging to the same speech utterance across streams. This may serve as a distributed clock: A binding mechanism that ensures the unified processing of different components of speech (Giraud and Poeppel, 2012; Yi et al., 2019) that are instead coded in a distributed fashion. Such a temporal code can also underlie binding of auditory sources in stream segregation (Elhilali et al., 2009). While in the current implementation of the model the responses are driven by stimuli, the model could be extended to include stimulus-independent oscillatory cortical activity. As the oscillations inherent to AC processing that occur on multiple timescales are known to decode complimentary informational structures in speech processing (Overath et al., 2015) and auditory scene analysis, such a model extension may in the future be used to study the effects on these ‘inherent’ oscillations on responses to speech and other structured inputs.

To summarize, we have presented a recurrent neural model built on simple and established assumptions on general mechanisms of neuronal processing and on the auditory cortical hierarchy. Despite its simplicity, the model was able to mimic results from (animal) electrophysiology and was useful to link these results to those of psychophysics and neuroimaging studies in humans. As the response properties of the AC (tonotopic organization, phase-locking, etc.) are inherited from the periphery, it remains possible that the model actually depicts earlier stages in the auditory pathway rather than AC. In future implementations of the model, the distinction between peripheral and cortical stages can benefit from a more detailed peripheral model (Meddis et al., 2013; Zilany et al., 2014). Ultimately, establishing a clear distinction between peripheral and cortical contribution would require simultaneous high-resolution (spatial and temporal) recordings across multiple locations of the auditory pathway and cortex. Furthermore, how the model dynamics shape up in presence of intrinsic noise in the system can also provide interesting insights into sound processing.

Nonetheless, the model is valuable for generating hypotheses on how the different cortical areas/streams may contribute toward behaviorally relevant aspects of acoustic signals. The presented model may be extended to include a physiological model of neurovascular coupling (Havlicek et al., 2017) and thus generate predictions that can be directly verified using functional MRI. Such a combination of modeling and imaging approaches is relevant for linking the spatially resolved but temporally slow hemodynamic signals to dynamic mechanisms of neuronal processing and interaction.

## Data Availability Statement

The datasets generated for this study are available on request to the corresponding author.

## Author Contributions

IZ and EF designed the model. IZ wrote the manuscript. All authors analyzed the model output. The manuscript was reviewed and edited by all authors.

## Conflict of Interest

The authors declare that the research was conducted in the absence of any commercial or financial relationships that could be construed as a potential conflict of interest.
