# Supplementary material for: Spectro-Temporal Processing in a Two-Stream Computational Model of Auditory Cortex
Source: Front Comput Neurosci. 2020 Jan 22;13:95. doi: 10.3389/fncom.2019.00095 (PMC6987265; doi:10.3389/fncom.2019.00095)
Supplement: Supplementary file 1 [file Data_Sheet_1.PDF]

Supplementary Material

1.1 Supplementary Figures

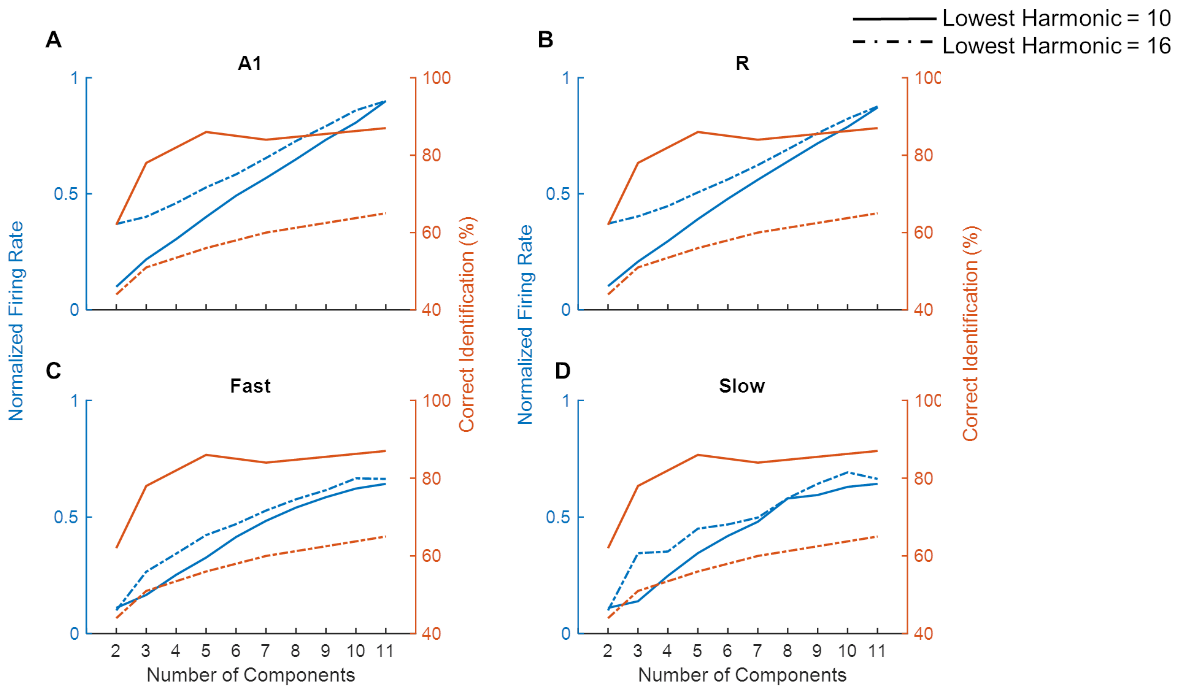

Supplementary Figure 1: Model performance on a missing fundamental task. The model performance in detecting missing fundamental of complex tones (average firing rate, normalized across all tones) is shown for areas A1, R, Fast and Slow (blue lines in panels A, B, C, and D respectively). Human behavioral data on pitch identification (%) task (Houtsma and Smurzynski, 1990) is plotted in orange lines. Solid lines show complex tones with lowest harmonic at 10 while the dash-dotted lines show lowest harmonic component at 16.
